# Evidence for colorectal cancer cell specificity of aspirin effects on NF*κ*B signalling and apoptosis

**DOI:** 10.1038/sj.bjc.6601913

**Published:** 2004-06-08

**Authors:** F V N Din, M G Dunlop, L A Stark

**Affiliations:** 1 Colon Cancer Genetics Group, University of Edinburgh Department of Oncology and MRC Human Genetics Unit, Western General Hospital, Crewe Rd, Edinburgh EH4 2XU, Scotland

**Keywords:** I*κ*B, NF*κ*B, NSAIDs, chemoprevention, colorectal cancer

## Abstract

Epidemiological evidence indicates that non-steroidal anti-inflammatory drugs (NSAIDs) protect against colorectal cancer (CRC) to a greater degree than other non-gastrointestinal cancers, but the molecular basis for this difference is unknown. We previously reported that aspirin induces signal-specific I*κ*Bα degradation followed by NF*κ*B nuclear translocation in CRC cells, and that this mechanism contributes substantially to aspirin-induced apoptosis. Here, we explored the hypothesis that cell-type specific effects on NF*κ*B signalling are responsible for the observed differences in protection by aspirin against CRC compared to breast and gynaecological cancers. We also assessed whether COX-2 expression, mutation status of adenomatous polyposis coli (APC), *β*-catenin, p53, or DNA mismatch repair (MMR) genes in CRC lines influenced aspirin-induced effects. We found that aspirin induced concentration-dependent I*κ*B*α* degradation, NF*κ*B nuclear translocation and apoptosis in all CRC lines studied. However, there was no such effect on the other cancer cell types, indicating a considerable degree of cell-type specificity. The lack of effect on NF*κ*B signalling, paralleled by absence of an apoptotic response to aspirin in non-CRC lines, strongly suggests a molecular rationale for the particular protective effect of NSAIDs against CRC. Effects on NF*κ*B and apoptosis were observed irrespective of COX-2 expression, or mutation status in APC, *β*-catenin, p53 and DNA MMR genes, underscoring the generality of the aspirin effect on NF*κ*B in CRC cells. These findings raise the possibility of cell-type specific targets for the development of novel chemopreventative agents.

Colorectal cancer (CRC) is common in developed countries (Parkin *et al*, 1999) and is a major contributor to cancer-related morbidity and mortality. Chemoprevention is an inherently appealing approach to combat the disease, and non-steroidal anti-inflammatory drugs (NSAIDs) have been associated with a substantial reduction in CRC incidence and mortality (Thun *et al*, 1993; Collet *et al*, 1999; Langman *et al*, 2000). Combined case–control data, including over 30 000 CRC cases, indicate a 45% reduction in the risk of developing CRC in subjects taking NSAIDs. Although there is evidence for a protective effect of NSAIDs against non-gastrointestinal cancers, the data are less convincing and the risk reduction much less. In breast cancer, reports show conflicting results and a recent meta-analysis revealed a risk reduction of only 13% in case–control studies (Khuder and Mutgi, 2001), considerably lesser than that in CRC. Similarly, in endometrial and ovarian cancer, the available evidence suggests that NSAIDs confer little, if any, protection (Cramer *et al*, 1998; Rosenberg *et al*, 2000; Fairfield *et al*, 2002; Meier *et al*, 2002). Collectively, published data suggest that there is considerable heterogeneity of NSAID anti-tumour effect *between* cancer types. The particular protective effect against CRC suggests the possibility that aspirin might target distinct molecular pathways in colonic epithelial cells. Elucidation of the molecular mechanism of this apparent differential sensitivity would lend further insight into both the mode of action of NSAIDs as well as identification of molecular markers of response.

The anti-tumour activity of NSAIDs has primarily been attributed to inhibition of the cyclooxygenase-2 enzyme (COX-2) and the resultant decrease in production of prostaglandins, as this remains the best-characterised effect (Vane, 1971). However, accumulating evidence from animal and cell culture experiments has shown that COX-2 inhibition is not the sole basis of NSAID anti-tumour activity (Alberts *et al*, 1995; Hanif *et al*, 1996; Elder *et al*, 1997; Piazza *et al*, 1997), suggesting that other targets are also involved. We previously reported that aspirin activates the NF*κ*B signalling pathway and that this mechanism is of central importance to aspirin-mediated apoptosis in CRC cells (Stark *et al*, 2001). The NF*κ*B transcription factor is normally sequestered in the cytoplasm by an inhibitor protein, I*κ*B*α*. Following stimulation of the NF*κ*B pathway, I*κ*B*α* is phosphorylated, ubiquitinated and targeted for proteosomal degradation. Dissociation from I*κ*B*α* results in translocation of NF*κ*B to the nucleus, where it contributes to the co-ordinated transcription of genes involved in inflammation, cell proliferation and apoptosis (Pahl, 1999). Our previous work demonstrated that aspirin induces time- and dose-dependent signal-specific degradation of I*κ*B*α*, nuclear translocation of NF*κ*B and apoptosis in CRC cells. Time-course experiments indicated that I*κ*B*α* degradation and NF*κ*B nuclear translocation preceded cell death, suggesting a causal relationship. This was confirmed in cells we engineered to continuously express a dominant-negative mutant I*κ*B*α* (I*κ*B*α*S32/36), which showed inhibition of both aspirin-induced NF*κ*B nuclear translocation and apoptosis compared to their parental counterparts (Stark *et al*, 2001). This work alluded to the notion of specificity since the NF*κ*B response was not observed in the control cell lines 293 HEK and A549, which were non-colorectal in origin.

Here, we focus on the important issue of the specificity of aspirin's protective effects, as observed in epidemiological studies, and we set out to determine whether cell-type specific effects on the NF*κ*B signalling pathway reflect the differential protective effects of aspirin in different cancer types. In particular, we wished to determine whether the lower protective effect observed for breast, ovarian and endometrial cancer can be explained by differing effects on the NF*κ*B signalling pathway. We also investigated the generality of the NF*κ*B response to aspirin in CRC by studying a panel of CRC cell lines with different genetic defects common in bowel malignancy. Here, we present evidence showing clear differences in NF*κ*B response that parallel the epidemiological data, supporting the notion that the ability of aspirin to modulate the NF*κ*B signalling pathway is a key determinant of the anti-tumour effect and that this is cell-type specific. Our findings provide further insight into the complex mechanisms by which NSAIDs exert an anti-tumour effect in CRC cells, and raise the possibility of cell-type specific molecular targets in CRC.

## MATERIALS AND METHODS

### Cell line culture and treatment

The CRC cell lines used were HRT-18, SW480, HT-29, DLD-1, LoVo and HCT116; breast cancer lines were T47-D, MCF-7 and MDA-MB-231; ovarian cancer line was A2780 and endometrial cancer line was HEC-1-A. All cancer cell lines are available from the American Type Culture Collection. The mutation status for the adenomatous polyposis coli (APC), p53, *β*-catenin and DNA mismatch repair (MMR) genes of the cell lines studied is shown in Table 1

**Table 1:** Mutation status of cancer cell lines studied

**Cell line**	**APC**	***β*-catenin**	**p53**	**MMR**
HRT-18	Mutant	Wild type	Mutant	Deficient
SW480	Mutant	Wild type	Mutant	Proficient
HT-29	Mutant	Wild type	Mutant	Proficient
DLD-1	Mutant	Wild type	Mutant	Deficient
LoVo	Mutant	Wild type	Wild type	Deficient
HCT-116	Wild type	Mutant	Wild type	Deficient
MCF-7	Wild type	Wild type	Wild type	Proficient
MDA-MB231	Wild type	Wild type	Mutant	Not known
T47D	Wild type	Wild type	Mutant	Proficient
A2780	Wild type	Wild type	Wild type	Proficient
HEC-1-A	Not known	Not known	Not known	Deficient

. Cell lines were grown as monolayers (37°C in 5% CO_2_) in RPMI (HRT-18, DLD-1 and A2780), DMEM (HT-29, T47-D, MCF-7, MDA-MB-231, HEC-1-A), L-15 (SW480) and McCoy's 5A media (HCT116) supplemented with 10% foetal calf serum (FCS) and 1% penicillin/streptomycin (media supplied by Gibco BRL, Paisley, UK). Cells were plated (1 × 10^6^ cells/50 ml flask) and grown until 60–70% confluent, prior to treatment with aspirin or carrier control at the same concentrations as the aspirin treatment. Aspirin (Sigma, St Louis, USA) was prepared as a 0.5 M stock solution in distilled water (final pH 7.0). Growth medium was replaced with the respective low serum (0.5% FCS) medium and cells were treated with aspirin at 1, 3, 5 and 10 mM for 24 h (or 72 h), or with carrier as a control.

### Cell viability and determination of apoptosis

Adherent cells were harvested and viable cell number determined by haemocytometric counts with nigrosin exclusion. IC_50_ values for the CRC cell lines were calculated using the XL*fit* 3™ software. Apoptosis was detected via its interaction with annexin V using an Annexin V-FITC apoptosis detection kit (Oncogene Research Products, Cambridge, MA, USA), as per the manufacturer's instructions. Briefly, the medium from the flask of adherent cells was transferred to a conical tube on ice to harvest any floating cells. Cells were then washed with 2 ml of PBS, which was also added to the tube to collect any cells dislodged during washing. Cells were incubated with 1 ml of trypsin : versene (volume per volume) just until the cells detached and then resuspended in the conical tube containing the media with the floating and washed cells. Cells were counted using a haemocytometer and resuspended in cold 1 × binding buffer to approximately 1 × 10^6^ cells ml^−1^. Media-binding reagent (10 *μ*l) was added to 0.5 ml of the cell suspension, which was incubated with 1.25 *μ*l of annexin V-FITC for 15 min at room temperature in the dark. Annexin V was then removed by centrifugation at 1000 *g* for 5 min and the cells were resuspended in 0.5 ml of cold 1 × binding buffer and placed on ice. The counting was done using a haemocytometer (two counting grids) in duplicate and this was carried out immediately following staining of cells, as apoptosis is an ongoing process and the FITC signal may be lost after an hour.

### Western blotting

Cells were washed with PBS, centrifuged (1200 r.p.m., 10 min) and cell pellets resuspended in lysis buffer (50 mM NaCl, 10 mM HEPES, 500 mM sucrose, 1 mM EDTA, 0.5 mM spermidine, 0.15 mM spermine, 0.2% Triton X-100) containing complete Protease Inhibitor Cocktail and 100 mM Pefabloc (Roche Diagnostics, Manheim, Germany). The cell suspension was centrifuged (6000 r.p.m., 15 min, 4°C) and the supernatant containing cytoplasmic proteins aliquoted. Protein content was measured by the method of Bradford (BioRad, Hercules, California, USA). Cytoplasmic proteins (30 *μ*g) were separated on a 10% SDS–PAGE gel, transferred to a polyvinylidine difluoride membrane (BioRad) and blocked in 4% non-fat dry milk solution with 0.3% Tween20 (Sigma). Membranes were probed with a sheep polyclonal I*κ*B*α* antibody (a gift from Professor R Hay, University of St Andrews, UK), rabbit polyclonal p65 antibody (Santa Cruz, California, USA) or mouse monoclonal COX-2 antibody (Cayman Chemicals, Michigan, USA). COX-2 electrophoresis standard (Cayman Chemicals) was used to indicate the correct COX-2 band. Monoclonal antibody to Cu/Zn SOD (The Binding Site, Birmingham, UK) and to actin (Santa Cruz) was used as a control for protein loading. Antigen–antibody complexes were visualised with chemiluminescence (Amersham ECL Reagents, UK).

### Immunofluorescence analysis

Cells grown to 60–70% confluence on glass coverslips were treated with carrier or 10 mM aspirin for 24 h (in the respective 0.5% FCS medium). After treatment, cells were washed with PBS, fixed with acetone : methanol (volume per volume) (−20°C, 10 min) and blocked in 10% pre-immune donkey serum (Sigma) for 1 h. Rabbit polyclonal antibody to NF*κ*B p65 (Santa Cruz) was applied for 1 h, followed by incubation with FITC-conjugated donkey anti-rabbit IgG for 1 h. The nuclei were stained with DAPI and the coverslips mounted with Vectashield (Vector Laboratories, Burlingame, California, USA).

## RESULTS

### Colorectal cancer cells are more susceptible to aspirin-induced apoptosis than non-CRC cells

We studied the effect of aspirin on the growth of a panel of CRC cell lines (HRT-18, SW480, HT-29, DLD-1, LoVo and HCT116) in comparison to cell lines derived from other cancer types: breast (MCF-7, MDA-MB-231, T47D), ovarian (A2780) and endometrial (HEC-1-A). The non-CRC cell lines were chosen based on epidemiological data, where there is some evidence to suggest a protective effect in breast cancer and less so in ovarian and endometrial cancer.

**Figure 1:**
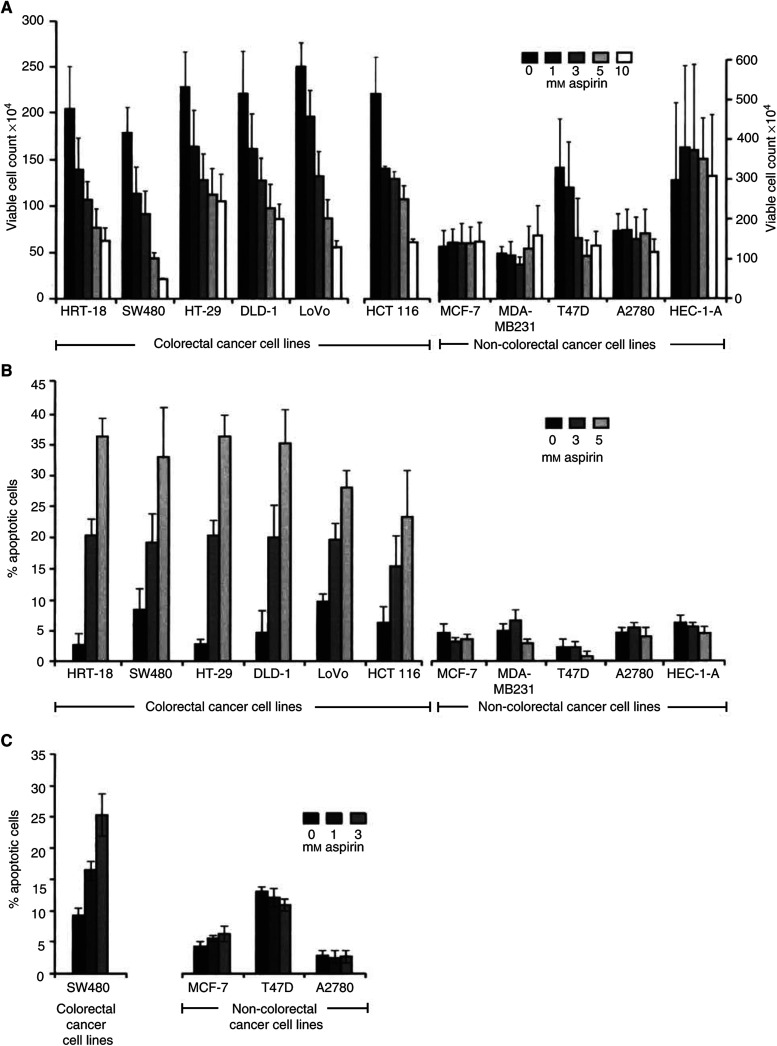
Differential effect of aspirin on cell viability and apoptosis in CRC and non-CRC cell lines. Aspirin treatment (0–10 mM) for 24 h induces a concentration-dependent decrease in viable cell number (determined by haemocytometric counts) in all CRC cell lines, but there is no consistent change in the non-colorectal cancer cell lines (**A**). Annexin V binding assay is used to determine whether all CRC cell lines undergo apoptosis after aspirin treatment (0–5 mM) for 24 h, but there was no change in apoptosis in the non-CRC cell lines (**B**). Annexin V binding assay is used to determine whether the non-CRC cell lines are less susceptible to aspirin-induced apoptosis compared to the CRC cell line SW480 following treatment for 72 h with aspirin (0–3 mM) (**C**). The graphs represent three independent experiments and the bars on the graphs are standard error bars.

In triplicate dose–response experiments, cell lines were treated for 24 h with aspirin at concentrations of 1, 3, 5 and 10 mM, and viable cell number determined by haemocytometric counts. We found a concentration-dependent decrease in viable cell number in each of the six CRC cell lines studied (Figure 1A

). In contrast, there was no demonstrable effect of aspirin on the viability of the non-CRC cell lines MCF-7, MDA-MB-231, A2780 and HEC-1-A (Figure 1A). Interestingly, the T47D breast cancer cells did exhibit a dose-dependent reduction in viability, although this effect was not so pronounced as that seen in CRC cells at low aspirin concentrations. The IC_50_ values were calculated from the growth curves of the aspirin-treated CRC cell lines only, as there was no consistent reduction in cell viability in the non-CRC cell lines (Table 2

**Table 2:** IC_50_ values for colorectal cancer cell lines

**Cell line**	**IC_50_**
HRT-18	3.12±0.69
SW480	1.48±0.12
HT-29	1.98±0.68
DLD-1	2.92±0.58
LoVo	2.07±0.25
HCT-116	2.71±0.46

). The mean IC_50_ value for the CRC cell lines was 2.38 mM and the greatest incremental reduction in viability in these cells was observed between 0 and 1 mM concentrations, which is comparable to serum concentrations attainable in humans (Pachman *et al*, 1979).

We next wished to establish whether the reduction in viable cell number that we observed in the CRC cell lines was due to induction of apoptosis. Annexin-V binding of phosphatidylserine residues externalised during apoptosis was used to determine the proportion of cells undergoing programmed cell death in response to increasing concentrations of aspirin. We found that aspirin treatment induced a concentration-dependent increase in apoptosis in all six of the CRC cell lines studied, confirming that induction of apoptosis is responsible for the observed reduction in cell viability (Figure 1B). There was no dose-dependent increase in apoptosis in the non-CRC cell lines following aspirin treatment, which was consistent with the lack of effect on cell viability (Figure 1A). Although the T47D breast cancer cells did exhibit a reduction in viable cell count, this effect was less marked than that seen in CRC cells and, furthermore, there was no increase in apoptosis in this cell line. To confirm that the non-CRC cells were less sensitive to apoptosis, we treated three non-CRC cell lines (two breast and one ovarian) and one CRC cell line (SW480) with aspirin for a longer time period of 72 h. Indeed, the non-CRC cell lines were far less susceptible to apoptosis compared to the CRC cell line despite treatment with aspirin for 72 h (Figure 1C). These findings demonstrate that the anti-tumour activity of aspirin has a substantial degree of specificity for CRC cells *in vitro*, reflecting the epidemiological evidence for a greater protective effect against CRC compared to other cancer types.

We also considered whether defects in genes commonly mutated in CRC, and known to affect apoptotic pathways, might influence such cell death. Mutation status for APC, *β*-catenin, p53 and DNA MMR genes (Table 1) does not appear to influence aspirin-induced apoptosis in CRC lines, emphasising the relevance of the aspirin NF*κ*B anti-tumour effect to CRC in general.

### Differential sensitivity to the apoptotic effects of aspirin is paralleled by differing responses of the NF*κ*B pathway

**Figure 2:**
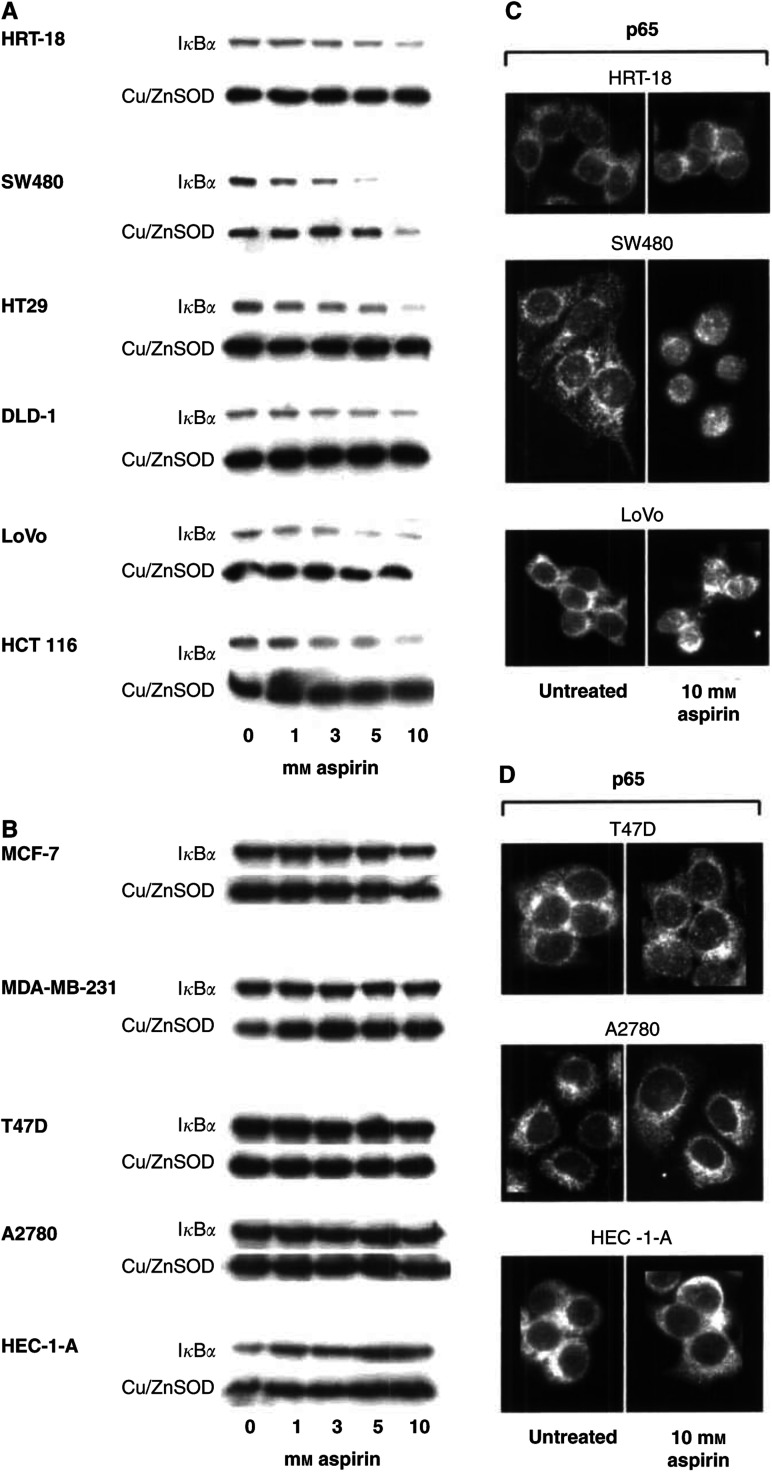
Aspirin-induced I*κ*B*α* degradation and p65 nuclear translocation is restricted to CRC lines. Western blot analysis shows that aspirin treatment (0–10 mM) for 24 h induces I*κ*B*α* degradation in a concentration-dependent manner in the CRC cell lines (**A**), but not in the non-CRC cell lines (**B**). Following aspirin treatment, cytoplasmic extracts were made from untreated and treated cells and probed with sheep polyclonal I*κ*B*α* antibody. The western blot shown is representative of at least three independent experiments, and Cu/Zn SOD was used as a control for protein loading. Micrographs (× 63) of immunocytochemically stained cells show that aspirin treatment (10 mM) for 24 h induces nuclear accumulation of p65 in the CRC cell lines (**C**), but not in the non-CRC cell lines (**D**).

Our previous work indicates that NF*κ*B nuclear translocation is a key component of aspirin-induced apoptosis in CRC cells. We therefore considered whether the variations in cell viability, observed between CRC and non-CRC cell lines, were attributable to differing responses of the NF*κ*B pathway to aspirin. We first investigated the effect of aspirin on cytoplasmic levels of the NF*κ*B inhibitor protein I*κ*B*α*, using immunoblot analysis. We found that aspirin treatment resulted in concentration-dependent degradation of I*κ*B*α*, as indicated by a reduction in cytoplasmic I*κ*B*α* protein levels (Figure 2A

) in all CRC cell lines. Although the I*κ*B*α* degradation may be more obvious at the higher doses, there is degradation at the lower doses of 1 and 3 mM aspirin (Figure 2A). The IC_50_ values for the CRC cell lines range from 1.48 to 3.12 mM aspirin, demonstrating differential sensitivity to aspirin with respect to the concentration at which 50% of the cells are growth inhibited (Table 2). The SW480 and HT-29 cell lines have IC_50_ values at the lower end of the range (1.48 and 1.98 mM, respectively) and do undergo I*κ*B*α* degradation at 1 mM, whereas the HRT-18 and DLD-1 cell lines have IC_50_ values of 3.12 and 2.92 mM and also exhibit I*κ*B*α* degradation at 3 mM. Hence, there is a close relationship between IC_50_ values and I*κ*B*α* degradation for the individual CRC cell lines. In striking contrast, there was no change in I*κ*B*α* levels upon aspirin treatment in any of the non-CRC cell lines even at the highest dose of 10 mM (Figure 2B).

Since these findings suggested a cell-type specific NF*κ*B response to aspirin, we next determined whether the disparate I*κ*B*α* response was accompanied by a differential effect on NF*κ*B nuclear translocation in the CRC compared to the non-CRC cell lines. Immunofluorescence analysis showed that p65, the transcriptionally active subunit of NF*κ*B, was primarily located in the cytoplasm in untreated cells as expected (Figure 2C, D, first panel). Following aspirin treatment, there was nuclear accumulation of p65 in all of the CRC cells (Figure 2C, second panel). However, in keeping with our observation that there was no I*κ*B*α* degradation in the non-CRC cells, aspirin treatment did not induce nuclear translocation of p65 in any of these cell lines (Figure 2D, second panel). These data establish that the disparity in viability following exposure to aspirin in CRC lines compared to lines derived from other cancer types is associated with markedly differing responses of the NF*κ*B pathway to aspirin. This work suggests that the effect of aspirin on NF*κ*B signalling may be implicated in the differential sensitivity of cancer types to aspirin-induced apoptosis.

### Basal I*κ*B*α* and p65 protein levels and aspirin-induced apoptosis in CRC cell lines

**Figure 3:**
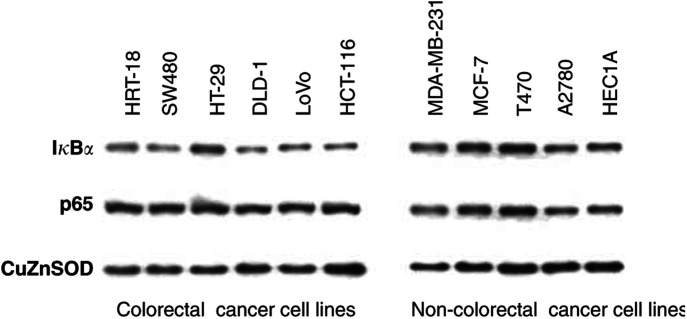
Western blot analysis demonstrates basal expression levels of cytoplasmic I*κ*B*α* and p65 proteins in CRC and non-CRC cell lines in untreated cells. Cytoplasmic extracts were made from untreated cells and probed with sheep polyclonal I*κ*B*α* antibody and rabbit polyclonal p65 antibody. The western blot shown is representative of at least three independent experiments and Cu/Zn SOD was used as a control for protein loading.

High basal NF*κ*B activity and aberrant I*κ*B*α* expression have been observed in a number of cancers including CRC (Rayet and Gelinas, 1999). In view of our findings of a cell-type specific NF*κ*B and death response to aspirin, we considered whether the basal levels of I*κ*B*α* and p65 might determine increased sensitivity to apoptosis, and so could be potential molecular markers of response. We used immunoblot analysis of cytoplasmic extracts to examine basal levels of I*κ*B*α* and p65 in both the CRC and non-CRC cell lines (Figure 3

). There was no difference in expression of I*κ*B*α* or p65 or their relative levels (analysed by densitometry, data not shown) between colorectal and non-CRC cells that could account for increased sensitivity to apoptosis. These results indicate that sensitivity to aspirin-induced apoptosis is not related to the cytoplasmic pool of either protein available for stimulation.

### Basal COX-2 protein levels do not determine the NF*κ*B response to aspirin

**Figure 4:**
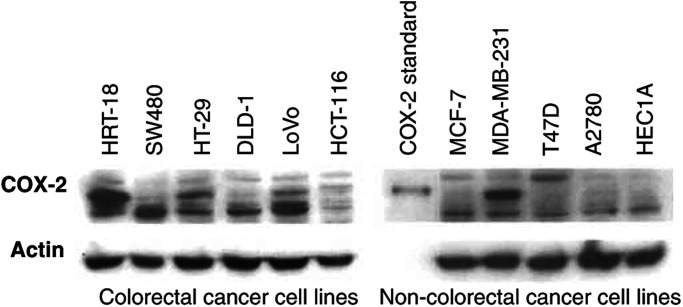
Western blot analysis of basal expression levels of cytoplasmic COX-2 protein in CRC and non-CRC cell lines. Cytoplasmic extracts were made from untreated cells and probed with mouse monoclonal COX-2 antibody. A COX-2 electrophoresis standard (72 kDa monomer) is used to indicate the COX-2 band. COX-2 is expressed in the HRT-18, HT-29, LoVo and MDA-MB-231 cell lines. The Western blot shown is representative of at least three independent experiments and actin was used as a control for protein loading.

Increased COX-2 expression has been observed both in premalignant colonic lesions and CRCs (Eberhart *et al*, 1994), and COX-2 inhibition has been shown to play a role in aspirin-mediated cell death (Boolbol *et al*, 1996). Hence, we considered whether COX-2 expression might explain the heterogeneity of the aspirin response between the CRC and non-CRC cell lines. Immunoblot analysis of cytoplasmic proteins demonstrated considerable variation in basal levels of COX-2 between the CRC cell lines (Figure 4

). The CRC cell lines SW480 and HCT116 do not express COX-2, whereas HT-29 and LoVo do express COX-2, and yet all underwent apoptosis following aspirin treatment. In the non-CRC panel, the MCF-7 cell line does not express COX-2 but the MDA-MB-231 cell line does express COX-2, but neither undergoes aspirin-induced apoptosis. Similarly, there was variability of COX-2 levels between the CRC lines and the non-CRC lines (Figure 4). Thus, we found no association between basal levels of COX-2 expression and sensitivity to aspirin-induced apoptosis, providing further support for the notion that COX-independent mechanisms play an important role in the anti-tumour effect of NSAIDs.

## DISCUSSION

The work presented here demonstrates a striking difference in the response to aspirin between CRC cell lines and lines derived from other cancer types, with respect to both cell viability and NF*κ*B signalling. We show that aspirin-induced apoptosis, associated with I*κ*B*α* degradation and NF*κ*B nuclear translocation, was restricted to CRC cells. This relationship between aspirin-induced apoptosis and the effect on NF*κ*B signalling suggests a molecular rationale for the particular sensitivity of CRC to NSAIDs compared to other cancers. These findings also extend our previous observations on the importance of the NF*κ*B pathway as a key NSAID target.

Epidemiological evidence indicates that NSAIDs impart greater protection against CRC than other cancer types, but the molecular basis for this effect is not known. Several previous reports, including our own, have shown that aspirin induces apoptosis in CRC cells (Hanif *et al*, 1996; Elder *et al*, 1997; Piazza *et al*, 1997; Qiao *et al*, 1998; Castano *et al*, 1999; Stark *et al*, 2001). There is little data directly comparing the anti-tumour effects of NSAIDs *in vitro* between CRC cells and cancer cells of different tissue origin. A recent study has demonstrated a tissue type-independent effect in prostate, lung, colon, tongue and pancreatic cancer using nitric oxide-donating NSAIDs and, although treatment with conventional NSAIDs did have a growth-inhibitory effect, it was observed at concentrations in excess of the pharmacologically relevant range after 48 h of treatment (Kashfi *et al*, 2002). Our findings demonstrate that aspirin has a considerable degree of specificity of apoptotic effect for CRC cells compared to other cell lines studied, and this reflects the epidemiological observations in the respective tumours. We show that aspirin induces apoptosis in a panel of CRC cell lines, but has no consistent effect on viability and apoptosis in cancer cell lines of non-colorectal origin. These results contrast with some previous reports of NSAID-induced growth inhibition and apoptosis in breast and endometrial cancer cells (Noguchi *et al*, 1995; Planchon *et al*, 1995; Han *et al*, 1998; Arango *et al*, 2001), but these differences are reconciled by considering that these studies used NSAIDs other than aspirin (Noguchi *et al*, 1995; Planchon *et al*, 1995; Han *et al*, 1998), while others only observed apoptosis after long exposures (48–96 h) to high concentrations of salicylate out with the therapeutic range (Sotiriou *et al*, 1999; Arango *et al*, 2001). The non-CRC cell lines are susceptible to other apoptosis-inducing agents and NF*κ*B activators such as staurosporine and TNF*α*, respectively, indicating that these cell lines are not generally resistant to apoptosis or NF*κ*B modulation (Mooney *et al*, 2002; Tang *et al*, 2002). The observation that aspirin decreased cell viability in one of the three breast cancer cell lines (T47D) is in keeping with epidemiological data that suggest a lesser protective effect of NSAIDs against breast cancer. One cohort epidemiological study showed that the effect of aspirin use on CRC incidence was reduced in females (Schreinemachers and Everson, 1994), raising the possibility that differential protection may be related to gender. However, gender is unlikely to impart a predominant protective effect, as it has not been borne out by subsequent studies (Thun *et al*, 1993). Indeed, the HT-29 CRC cell line is derived from a female patient and is equally susceptible to aspirin-mediated apoptosis and NF*κ*B modulation as the other CRC cell lines, which are male in origin. Our findings clearly indicate important differences between CRC and other cancer types with respect to aspirin effects on cell viability and apoptosis.

We show that aspirin-induced apoptosis occurs following I*κ*B*α* degradation and NF*κ*B nuclear translocation, and that this effect is common to all CRC cell lines studied. Notably, this effect on the NF*κ*B pathway was consistent between CRC cell lines despite heterogeneity of the lines, with respect to the profile of mutations in APC, *β*-catenin, p53 and DNA MMR genes (see Table 1). In contrast, aspirin treatment did not induce I*κ*B*α* degradation or NF*κ*B nuclear translocation in any cell lines derived from cancers of other tissue types, paralleling the lack of consistent changes in cell viability and apoptosis in these lines. We have previously established that the observed effect of aspirin on I*κ*B*α* and p65 is a *cause* of rather than a consequence of apoptosis, based on the findings that the I*κ*B*α* degradation was signal-specific and that nuclear translocation of NF*κ*B and apoptosis were blocked by a dominant-negative super repressor I*κ*B*α* (Stark *et al*, 2001). Furthermore, we showed that I*κ*B degradation and NF*κ*B nuclear translocation occur at 2–5 h after aspirin treatment and persists thereafter to 24 h, whereas apoptosis is not observed to increase until at least 16 h and continues to 24 h. Hence, the weight of evidence presented here correlating I*κ*B*α* degradation and p65 nuclear translocation with apoptosis compared to the lack of response in non-CRC cell lines provides considerable further support for a causal role of the NF*κ*B response as an important component of aspirin-induced apoptosis.

Having shown a striking difference between the CRC and non-CRC cell lines, with respect to aspirin effects on NF*κ*B signalling and apoptosis, we investigated potential factors that might contribute to the ability of specific cell types to undergo apoptosis. Increased NF*κ*B activity has been observed in CRC (Hardwick *et al*, 2001) and relative resistance to apoptosis has been attributed to high constitutive NF*κ*B activity in other cancers (Bours *et al*, 1994; Lind *et al*, 2001; Charalambous *et al*, 2003). However, we found no evidence that the specificity of the aspirin-NF*κ*B response is related to differential expression of basal I*κ*B*α* or p65 proteins or their relative expression. The SW480 and HT-29 CRC cell lines undergo NF*κ*B-mediated apoptosis, despite the considerable difference in basal NF*κ*B activity previously reported between these cell lines (Dejardin *et al*, 1999).

There is substantial rationale for investigating COX-2 as a potential molecular determinant of response, in view of its role in CRC development and as a pharmacological target for NSAIDs. Additionally, it has been reported that the inconclusive nature of epidemiological data in breast cancer might be related to the observation that only a subset of breast cancers express COX-2 (Howe *et al*, 2001). In the work presented here, we did not detect a relationship between COX-2 protein levels and apoptotic response to aspirin in any cell type. Furthermore, the fact that we observed considerable variation in COX-2 expression within the CRC cell lines, which were all susceptible to aspirin-induced apoptosis, presents persuasive evidence that COX-2-independent as well as COX-2-dependent mechanisms play a role in the anti-tumour effects of NSAIDs (Rigas and Shiff, 2000).

Aspirin concentrations used here are relevant to pharmacological levels in clinical practice (1–3 mM) (Insel, 1996). Nonetheless, comparisons between cell culture concentrations and plasma levels are somewhat artificial, because of the inability to accurately mimic *in vivo* metabolism and tissue concentration of the agent in epithelial or tumour cells. Decreased basal levels of apoptosis and hyperproliferative mucosa have been observed in patients with adenomas, suggesting the existence of a ‘field defect’ in the colonic mucosa (Anti *et al*, 2001). Although we observed proportionally less apoptosis at lower concentrations of aspirin, there is evidence that low levels of apoptosis translate into significant tumour regression over time in cell kinetics studies (Pritchard and Watson, 1996). It remains to be determined whether aspirin redresses the balance by inducing apoptosis *de novo* in newly transformed colorectal epithelial cells destined to become malignant clones. There is evidence of NF*κ*B involvement in colonic crypt differentiation and cell turnover in mouse colon, where NF*κ*B activity is greater in proliferating cells at the base of crypts compared to mature cells at the surface (Inan *et al*, 2000). Thus, it is also possible that the drug corrects deranged mechanisms that permit escape from normal cellular turnover and apoptosis.

In summary, the data presented here demonstrate that there are substantial differences in the anti-tumour effects of aspirin and modulation of NF*κ*B signalling between cancer cells of different tissue origin. The effect of aspirin on NF*κ*B signalling and apoptosis does not appear to be related to expression levels of COX-2 or mutation status of APC, *β*-catenin, p53 and DNA MMR genes. This is important when considering translating these findings to clinical studies aimed at defining the NF*κ*B response to aspirin in human colonic epithelium and tumours. The molecular basis of NSAID anti-tumour activity is complex, and our findings provide further evidence that the effects of aspirin on NF*κ*B signalling have particular relevance to CRC chemoprevention.
